# Fibromyalgia in Pregnancy: Neuro-Endocrine Fluctuations Provide Insight into Pathophysiology and Neuromodulation Treatment

**DOI:** 10.3390/biomedicines11020615

**Published:** 2023-02-18

**Authors:** Viviana Mucci, Ilaria Demori, Cherylea J. Browne, Choi Deblieck, Bruno Burlando

**Affiliations:** 1 School of Science, Western Sydney University, Campbelltown, NSW 2560, Australia; 2 Department of Earth, Environmental and Life Sciences (DISTAV), University of Genova, Corso Europa, 26, 16132 Genova, Italy; 3 Translational Neuroscience Facility, School of Medical Sciences, UNSW Sydney, Kensington, NSW 2052, Australia; 4 Brain Stimulation and Rehabilitation (BrainStAR) Lab, School of Health Sciences, Western Sydney University, Campbelltown, NSW 2560, Australia; 5 Antwerp Management School, University of Antwerp, Boogkeers 5, 2000 Antwerp, Belgium; 6 Department of Pharmacy, DIFAR, University of Genova, Viale Benedetto XV, 3, 16132 Genova, Italy

**Keywords:** chronic pain, gonadal hormones, cortisol, GABA, serotonin, tDCS

## Abstract

Fibromyalgia (FM) is a chronic pain disorder with unclear pathophysiological mechanisms, which leads to challenges in patient management. In addition to pain, the disorder presents with a broad range of symptoms, such as sleep disruption, chronic fatigue, brain fog, depression, muscle stiffness, and migraine. FM has a considerable female prevalence, and it has been shown that symptoms are influenced by the menstrual cycle and periods of significant hormonal and immunological changes. There is increasing evidence that females with FM experience an aggravation of symptoms in pregnancy, particularly during the third trimester and after childbirth. In this perspective paper, we focus on the neuro-endocrine interactions that occur between progesterone, allopregnanolone, and cortisol during pregnancy, and propose that they align with our previously proposed model of FM pathogenesis based on GABAergic “weakening” in a thalamocortical neural loop system. Based on our hypothesis, we introduce the possibility of utilizing transcranial direct current stimulation (tDCS) as a non-invasive treatment potentially capable of exerting sex-specific effects on FM patients.

## 1. Introduction

Fibromyalgia (FM) is a debilitating, chronic pain disorder with inconclusive underlying mechanisms. Due to the lack of understanding of its pathogenesis, treatment of this clinical population is challenging [1]. FM was originally classified as a peripheral musculoskeletal disorder; however, research has led to a redefinition of the syndrome as a central disorder involving pain processing [2], categorized as a Central Sensitivity Syndrome (CSS). Even though chronic widespread pain is the most well-known characteristic of FM, the disorder encompasses a broad range of symptoms, such as sleep disruption, chronic fatigue, depression, anxiety, and muscle stiffness. Moreover, it is associated with various comorbidities, such as migraine and irritable bowel syndrome [3,4], which both lie within the CSS category.

FM occurs in both males and females; however, females represent the majority of the FM clinical population [5,6,7], with peak occurrence at around postmenopausal ages [8]. In pre-pubertal children, FM incidence is low and does not demonstrate the same gender imbalance which occurs in adults. However, at the onset of puberty, FM rates become female dominant [9]. This suggests that gonadal hormones play a role in the development of FM, similarly to migraine [10]. The female reproductive cycle and pregnancy involve significant neuro-endocrine changes [11], which are considered potential contributors to FM pathogenesis [12,13]. Estrogens and progesterone are known to exert multiple non-reproductive influences on the central nervous system, including protective effects against glutamate excitotoxicity, amyloid beta, and oxidative stress, and in addition, the recovery from traumatic brain injuries [14,15,16,17]. These hormone fluctuations are thought to be one of the main mechanisms responsible for the higher prevalence of chronic pain conditions in females [18,19]. In addition, gonadal hormone fluctuations present in the life of women are associated with modifications in immunocompetence and symptomatology [20,21]. Animal research highlighted how neuro-immune pain signaling may have sexual dimorphisms [22,23]. FM has, therefore, been proposed as a neuropathy-induced, autoimmune syndrome where women’s peculiar endocrinological and immune responses might be accountable for a clear female predominance in the manifestation of the disorder [13,24].

In a previous paper, we formulated a hypothesis that FM pathophysiology depends on a switch in the functioning of a thalamocortical loop system, resulting in chronic pain [25]. The thalamocortical loop normally acts as a negative feedback loop due to GABAergic modulation exerted by the thalamic reticular nucleus on the reciprocal excitatory connections between the thalamic ventroposterolateral nucleus and the primary somatosensory cortex. GABA, the most common inhibitory neurotransmitter in the central nervous system [26], seems to play a crucial role in FM pathophysiology, particularly being implicated in FM-altered central pain circuitry [25,27]. To support this, it has been shown that individuals with FM have lower levels of GABA in brain regions responsible for sensory processing when compared to healthy controls [28]. In addition, anticonvulsant medications that modulate GABA, such as pregabalin and gabapentin, show some efficacy as FM treatment [29]. Also, immune-endocrine stimuli, such as menopause [30], activation of the stress response [31], and increased inflammatory cytokines [32], can be linked to the weakening of GABAergic transmission in FM [25]. In our model, if GABA is reduced, and/or glutamate rises, the thalamocortical neural loop system shifts to a bistable switch, predisposed to developing a high pain processing response after minimal or even absent peripheral stimuli (phantom stimuli), resulting in the chronic pain condition of FM [25]. This hypothetical model resolves various FM etiological correlations, neuroimaging evidence, and clinical data [25,33], thus suggesting GABA modulators as a preferential therapeutic option. Here we propose the use of non-invasive brain stimulation (NIBS) for its ability to modulate GABAergic activity.

Over the past 20 years, interest in NIBS, such as transcranial direct current stimulation (tDCS) and transcranial magnetic stimulation (TMS), has surged. Although the gender component has not been significantly considered in the implementation of neuromodulation, it has been shown that females with major depression are 1.34 times more likely to respond and 1.37 times more likely to achieve remission relative to males with tDCS treatment [34]. Similarly, a positive, linear relationship between the percentage of females enrolled in clinical trials, and the overall reduction in depression severity has been reported [35,36,37]. Compared to males, females have greater gray matter volume within the frontal and parietal cortices, cerebral blood flow, and baseline neural activity [38,39]. The frontal and parietal cortices of females are also associated with a higher gyrification index (a measurement of cortical folding), leading to an increase in the gyral surface area [40]. The variance in observed data may be related to the impact that fluctuations in estradiol and progesterone have on cortical excitability [41,42,43]. Therefore, we propose that the use of neuromodulation treatments for the management of a disorder such as FM might be effective considering peculiar hormonal phases, such as pregnancy, which is entirely novel.

Considering these aspects, the current perspective paper aims to highlight the mechanistic hypothesis on how the neuro-endocrine changes during pregnancy can influence our thalamocortical loop model and FM pathophysiology. It also proposes the use of tDCS as a therapeutic intervention based on its ability to modulate the GABAergic system [44].

## 2. Methods

The current manuscript is a perspective paper that presents a hypothesis about the role of gonadal hormones during pregnancy in FM patients. The hypothesis was developed based on a PubMed database literature search, from inception through November 2022 and was based on our previously published theoretical loop model [25]. During the PubMed search, primary endpoints were the combination between the terms “*fibromyalgia*” or “*pain*”, and in addition, the combinations of each of the former terms with a series of terms. These terms include: “*androgens*”, “*estrogens*”, “*GABA*”, “*glucocorticoids*”, “*glutamate*”, “*gonadal hormones*”, “*HPA axis*”, “*HPG axis*”, “*immunity*”, “*interleukins*”, “*neuromodulation*”, “*pregnancy*”, “*serotonin*”, “*transcranial direct current stimulation*”, “*transcranial magnetic stimulation*”, and *allied terms*. The literature review results were used in support of the new perspective hypothesis proposed in this manuscript.

## 3. Interactions between FM and Pregnancy

The literature on the interaction between FM and pregnancy is limited, though the available studies do provide evidence that justifies further exploration of this relationship. Two studies have reported that around 50% of their study population experienced FM symptoms after childbirth, and this rate was higher in those who had delivered by cesarians vs. vaginal births [45,46]. However, these studies did not confirm whether any of these patients had pre-existing FM. Other studies demonstrated that pregnant females with pre-existing FM experienced an aggravation of symptoms throughout pregnancy, and particularly in the third trimester, the symptoms were at their worst [46,47,48]. There were also reported changes to symptoms in the postnatal period, while hormonal changes connected with abortion, use of hormonal contraceptives, and breastfeeding did not modulate FM symptom severity [45,49,50]. Overall, these data highlight how neuro-endocrine fluctuations during pregnancy may exacerbate FM pain symptoms, similarly to what was also reported during menses [51,52]. Therefore, in the next paragraphs, we consider some of the major signaling molecules whose levels vary widely in pregnancy and highlight their possible role in FM pain modulation.

## 4. Estrogen, Progesterone, and Cortisol

Given that most FM patients are females, gonadal hormone influence has been widely investigated. Females affected by FM are experiencing more FM induced-pain during the luteal phase and the immediate time before and after menses, in comparison with healthy controls [51,52,53]. From clinical observations as well as animal models, we know that pain processing has been linked with changes in estrogen levels. Many studies have proven that estrogen modulates pain via specific signaling pathways. However, it is still unclear which subtype of estrogen receptor (ER) is recruited under different conditions [54]. Estrogen’s influence on pain remains controversial, with either pro-algesic or analgesic properties reported in different studies [19]. This is possibly because estrogens are not easily separated from other hormones, being preceded by an increase in testosterone and co-occurring with an increase in progesterone. A study on transgender people argues for pain aggravation, showing that 55% of female-to-male individuals with chronic pain reported a reduction of pain after receiving testosterone treatment, while 23% of male-to-female individuals reported initiation of chronic pain after estrogen and anti-androgen therapy [55]. By contrast, anti-nociceptive effects of estrogens have been reported in a mouse model of neuropathic pain [56]. Potentially, these differences are due to the activation of different ERs [54]. A study showed that ERβ agonists were effective in alleviating pain induced by chemotherapy, while the nonselective agonist 17β-estradiol and the ERα-selective agonist PPT had no effect [57].

Conversely to estrogens, the role of progesterone in pain modulation seems better defined. A study on pre-menopausal females with FM subjected the females to daily measurements of plasma hormone levels throughout the menstrual cycle. Results showed that progesterone and testosterone, but not estradiol or cortisol, were inversely correlated with pain severity, reporting that pain was highest during the menstrual phase when gonadal hormones were at their lowest levels [58]. Progesterone and neurosteroid allopregnanolone, a progesterone metabolite, are known to reduce neuropathic pain. This is due to their modulatory properties of GABAergic transmission, particularly their allosteric stimulation of the GABA_A_ receptors (GABA_A_R), as reported in animal model studies [59]. Despite considerable interpersonal variability, both estradiol and progesterone fluctuate, and their serum levels increase enormously during pregnancy up to the third trimester (see Table 1). We, therefore, consider the possible involvement of this surge of hormones in the variations of symptoms in FM patients.

Other important hormones to consider are glucocorticoids (cortisol in humans), which are essential to sustaining pregnancy. These hormones are involved in the development of the fetal organs and modulate the increased energy demand during the gestational period. Therefore, modifications of the hypothalamic–pituitary–adrenal (HPA) axis are part of the physiological adaptive mechanisms in pregnancy. Starting from the end of the first trimester, cortisol production is enhanced, reaching serum levels up to about 50 µg/dL at the end of gestation (Table 1). Placental corticotropin-releasing hormone contributes to this physiological hypercortisolism and plays a role in inducing labor [62,63]. Nevertheless, the fetus is protected from excess cortisol by means of different mechanisms, including the placental activity of type 2 11β-hydroxysteroid dehydrogenase, responsible for cortisol inactivation [64], and the downregulation of the maternal stress response. The latter is achieved through the induction of neurosteroidogenesis by pregnancy-associated hormones, such as estrogens, progesterone, and prolactin [65]. Particularly, the levels of allopregnanolone increase together with progesterone during pregnancy. Allopregnanolone stimulates GABAergic neurotransmission and the endogenous opioid system at the hypothalamic paraventricular nucleus to dampen down the HPA response to stressors [66].

Despite these protective mechanisms, cortisol levels can still rise in response to traumatic or chronic stress during pregnancy. In addition, according to the “pregnenolone steal” hypothesis, the enhanced synthesis of cortisol upon stress may reduce the amount of pregnenolone available for the synthesis of other steroids, leading to progesterone and allopregnanolone depletion [62]. While this hypothesis has yet to be verified, it provides a link between cortisol and progesterone that could explain the onset or worsening of FM in late pregnancy. In fact, reduced allopregnanolone bioavailability can impair GABAergic transmission. In addition, cortisol, even at lower concentrations than those activating glucocorticoid receptors, binds to the membrane or cytoplasmic mineralcorticoid receptors, which have been shown to act pre- and post-synaptically to facilitate glutamatergic transmission [67].

In addition to this body of evidence, it has been shown that the action of allopregnanolone at the highest physiological concentrations can involve a peculiar dose–response inversion, from activation to inhibition, of GABA_A_R, specifically the α_4_β_2_δ subunit combination [50,68]. This could further explain why FM symptoms worsen in late pregnancy when progesterone and allopregnanolone are at maximum levels before decreasing. Although this makes theoretical sense, the actual bioavailability of neurosteroids in the brains of pregnant females is still unknown, and further studies should be encouraged [65].

It is also worth noting that after a period of prolonged hypercortisolism, such as that occurring when high levels of stress add up to the physiological increase in cortisol during pregnancy, compensatory mechanisms may be triggered, including blunted HPA axis responsiveness and glucocorticoid resistance. In accordance, hypocortisolism has been observed in about one-quarter of patients with stress-related disorders, including FM [69,70].

## 5. Prolactin and Immune Mediators

Another hormone showing large variations during pregnancy is prolactin (PRL) (Table 1). High levels of estrogens stimulate the pituitary secretion of PRL throughout pregnancy, reaching peak serum values at term and delivery [71]. It has been shown that in vitro PRL affects hormonal secretion by placental cells: the secretion of progesterone is increased, whilst that of estrogens is decreased [72]. However, PRL is also among the hormonal mediators that are released during stress, while substantial evidence supports a stimulatory action of PRL in the adrenal gland’s response to stress [73]. Moreover, PRL has cytokine properties and immunostimulatory actions and promotes autoimmunity [74].

As for the role of PRL on neurotransmitters, it has been found that lactogenic induction of maternal behavior is mediated by PRL receptors expressed on GABAergic neurons [75]. Moreover, PRL is known to increase the synthesis and release of GABA in hypothalamic tissue [76]. Given the wide expression of prolactin receptors in the CNS [77], it is possible that PRL exerts a positive effect on different central GABAergic functions.

Besides canonical hormones, gestation is also a period of intense variations of immune mediators, some of which also act as signal molecules to regulate various physiological processes [78]. Overall, a three-phase sequence can be outlined in pregnancy, namely inflammatory/anti-inflammatory/inflammatory, which roughly corresponds to trimesters, with a more vigorous inflammatory phase in late pregnancy [79]. Successful fetal gestation relies on the crosstalk between the endocrine and immune systems. Understanding these complicated mechanisms is fundamental to our understanding of the potential neurophysiological changes that occur in FM patients [80]. These interactions are made possible by the expression of hormone receptors on a wealth of immune cells, and by the responsiveness of endocrine tissues to immune mediators, such as cytokines [81]. Although the role of cytokines in FM is still unclear, it should be considered when investigating the crosstalk between the endocrine and immune systems [82].

## 6. Neurotransmitters

Increasing evidence based on experimental models and brain imaging has demonstrated the anti-nociceptive effects mediated by the serotonin (5-HT) receptors 5-HT1A and 5-HT1B [30,31]. An interaction between estrogens and serotoninergic pathways seems to be mediated by the activation of genomic and non-genomic estrogen receptors (ERs). More specifically, 5-HT2A receptors have been shown to decrease pain when estrogen levels are high, while their blockage by a 5-HT2A antagonist tends to increase the estrogen-induced pain release [39,40]. Some reports on FM patients mention low 5-HT serum levels, which has been proposed as a potential FM biomarker [83]. Such a reduction could be associated with a reduction in the levels of melatonin, which partially explains these patients’ disturbed sleep patterns [84].

A clue about the possible involvement of low 5-HT in FM pathophysiology is offered by pharmacologic interventions based on 5-HT modulation, such as antidepressants (e.g., amitriptyline) [85]. Similarly, some non-pharmacologic FM therapies, such as acupuncture, could be related to an increase in 5-HT. Although the mechanism of action of acupuncture is unknown, the stimulation of acupoints can alter the concentration of some pain mediators, including endorphin, substance P, encephalin, and 5-HT, in the brain and local tissues [86,87]. However, the role of 5-HT in the descending pain regulatory pathway should be ruled out in FM. This is because the limited therapeutic effects of opioids show that this pathway could be, at most, only indirectly involved in the disorder [88].

However, 5-HT is also known to strengthen both excitatory glutamate and inhibitory GABA synapses [89,90]. Therefore, it potentially acts positively on the thalamocortical major supraspinal relay site for ascending pain stimuli. The role of GABA and 5-HT in modulating pain processing during pregnancy is still uncertain. In pregnant female rodents, a reduction of brain GABA levels and a downregulation of GABA_A_R δ and γ_2_ subunits have been found [91,92]. In pregnant females with FM, 5-HT has been shown to decrease as pregnancy progresses. This resulted in an increase in anxiety and depression [93], but may have also contributed to explaining pain recrudescence in late pregnancy [94].

## 7. Perspectives for a Validation of Our Hypothesis

**Figure 1:**
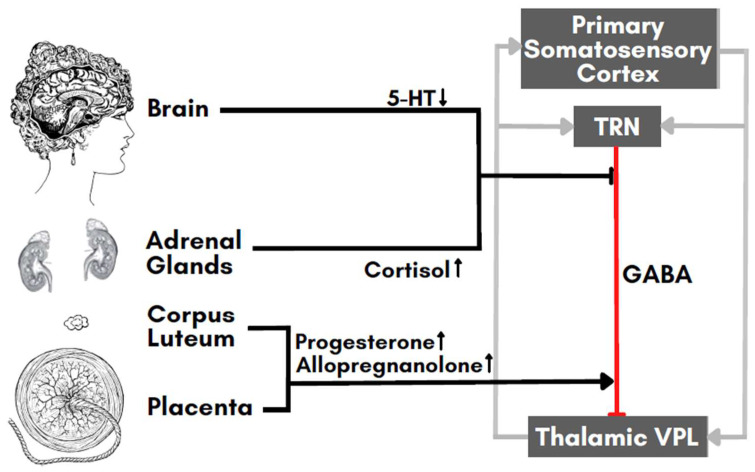
Effects of major pregnancy endocrine agents on the thalamocortical loop brain network representing a possible bistable switch model of FM pathogenesis. Progesterone and allopregnanolone strengthen GABAergic transmission, whereas cortisol indirectly weakens it, thereby preventing or promoting, respectively, the switching of the loop to a pathogenic functional regime. Key: Arrow line-endings indicate activation and T-shaped line-endings inhibition. TRN: thalamic reticular nucleus, VPL: ventroposterolateral nucleus. Image attributes: Henry Vandyke Carter and Pearson Scott Foresman, Public domain, via Wikimedia Commons https://commons.wikimedia.org (accessed on 17 February 2023).

Our FM model can combine into a consistent scenario some of the above-described neuro-endocrine mechanisms to explain FM pathophysiology during pregnancy (Figure 1). The role of estrogens in modulating pain is still controversial. Similarly, PRL could promote the GABAergic function, which, in our model, preserves from FM insurgence, but has also been shown to increase the HPA axis during stress, which is detrimental to GABA. Conversely, the role of progesterone seems better defined. It is widely accepted that progesterone is a major pain modulator in pregnancy, possibly through its neurosteroid allopregnanolone [95,96]. In addition, the role of 5-HT depletion in FM progression and pain symptoms recrudescence seems crucial [93].

Early-to-mid pregnancy corresponds to increasing systemic progesterone, and consequently, to high brain allopregnanolone, presumably involving a positive effect on GABAergic transmission. Conversely, in the third trimester, there are a few events to consider: (i) pregnenolone steal and glutamate pumping by cortisol, (ii) possible inversion of GABAergic transmission from inhibitory to excitatory induced by prolonged high allopregnanolone, and (iii) inflammation rebound. These events could collectively contribute to weakening the inhibitory GABAergic and/or strengthening the glutamatergic function, thus explaining FM symptom worsening and shifting the thalamocortical loop of our model towards a positive feedback loop [25].

Late pregnancy events can be exacerbated by a disequilibrium between glucocorticoids and progesterone production. This is due to maternal stress, which triggers glucocorticoid release and impairs progesterone secretion, causing inflammation [62]. As known, systemic inflammation can trigger neuroinflammation [97] and the latter is associated with excitotoxicity due to glutamate/GABA imbalance [98,99]. Finally, 5-HT lowering during pregnancy could also produce detrimental effects on thalamic GABAergic transmission. This is caused by a 5-HT modulatory effect on presynaptic activity [100]. Hence, the correlation between neuroendocrine variations and FM changes during pregnancy and postpartum are overall in agreement with the predictions of our thalamocortical loop model.

## 8. Future Interventions for Neuromodulation in Pregnant FM Patients

As we have highlighted, the immune-endocrine scenario of pregnancy helps to clarify the upstream mechanisms that would trigger the FM pathogenic transition as predicted by our model. These correspondences add to a series of pharmacological and clinical data that are consistent with the model [25], thus strengthening its reliability. Hence, given that the model involves a brain network dysfunction, neuromodulation techniques deserve particular attention in the development of a suitable therapeutic strategy for FM.

Proposed explanations for sex-mediated effects of brain stimulation on cortical excitability and behavior include: (i) neurotransmitter balances; (ii) cortical bone structure/composition; (iii) distance from the prefrontal cortex to the external surface of the skull; (iv) structural and functional differences; (v) anatomical differences in tissue volumes; (vi) gonadal hormones [101].

Considering these crucial sex-mediated effects of brain stimulation, it is interesting to further explore such techniques in a gender-driven disorder such as FM. Performing new studies on non-invasive techniques is necessary. Due to deep uncertainties about the pathogenesis and development of FM, the clinical management of patients is generally challenging and often provides limited results [102]. This becomes even more complicated in female patients during pregnancy. Different drugs commonly used in FM for managing pain and other symptoms are considered problematic for possible adverse gestational outcomes [94]. Antidepressants, together with gabapentinoids, are a first-line FM therapeutic choice [103], but when nursing, these drugs are typically restricted to the treatment of moderate to severe depression due to unknown long-term effects on the developing infant’s nervous system [104]. Such a complex of reasons raises the need for alternative therapeutic treatments during the perinatal period [105].

Utilizing neuromodulation techniques would also be a way to validate our theoretical model by inducing an effect on the GABAergic system. A major mechanism by which the neural activity generated by an experience modifies brain function is via modifications of synaptic transmission, known as synaptic plasticity. There is agreement that alterations of synaptic transmission are based on a delicate balance between excitatory and inhibitory processes [106,107]. Glutamate is the most common excitatory neurotransmitter in the central nervous system [26], while it is estimated that 30% of synapses in the mammalian cerebral cortex are GABAergic [108]. Hence, the homeostasis of the glutamate/GABA balance is crucial in the modulation of proper cortical excitability [109]. Considering the influence of tDCS on the GABAergic system, a potential approach would be to treat FM patients with neuromodulation prior to a planned pregnancy. Females have been reported to show enhanced response following NIBS compared to males [110,111]. Although this finding is not universal [112], non-invasive neuromodulation might be a promising alternative treatment option for pregnant FM patients [113].

Considering the hormonal differences between females and males, sex-specific neuromodulation protocols that consider different hormonal phases are essential in addressing gender-driven disorders such as FM. Testosterone and its metabolites modulate cortical excitability similarly on different days, as opposed to the cyclic fluctuations and, therefore, the effect female hormones have on cortical excitability. Particularly, when progesterone and estradiol are low during the first follicular phase of menstruation (days 1–7), cortical excitation and inhibition from tDCS are less responsive. As estradiol increases, while progesterone remains low in the second follicular phase (days 7–14), excitability is enhanced while inhibition is reduced. When estradiol levels are moderate and progesterone levels are high (during the first (days 14–21) and second luteal phase (days 21–28)), excitation is reduced and inhibition is enhanced [41,42].

In 2006, the first randomized, sham-controlled proof-of-principle study on tDCS provided initial evidence of the therapeutic effect of this technique in FM [114]. Today, based on published Pubmed-indexed data, approximately 80 studies report the use of tDCS as a treatment option in FM. It should be highlighted that effect size and duration are often limited in any disorder using tDCS [115]. Therefore, research into the probable effect of tDCS in FM is encouraged to make these studies part of routine clinical care. A self-administered tDCS in the home setting could be helpful to ease access to treatment [116]. When implementing neuromodulation protocols, it is important to consider the hormonal phase of females and the gender dimorphism with males. With regards to young female patients with FM planning to become pregnant, it is certainly easier to implement these protocols as part of a routine clinical practice prior to or after pregnancy. However, studies should further explore the usage of tDCS during pregnancy. In theory, tDCS poses no risk to a developing fetus when applied during pregnancy [112]. This is because tDCS changes regional brain activity without directly impacting autonomic or thermoregulatory functions [113,117]. Yet, more conclusive data are required.

To date, tDCS in pregnancy has only been tested in four studies (case report, uncontrolled, randomized clinical trial, open label) in Major Depressive Disorder (MDD) and two case reports in Auditory Hallucinations (AH) (Table 2). All six studies supported the potential benefit of tDCS in the treatment of MDD or AH during pregnancy. No serious pregnancy or birth complications or irregularities of fetal or maternal health were observed in more than 120 tDCS sessions [112], or 160 tDCS sessions [118]. Minimum anticipated side effects were reported, and patients were overall satisfied using tDCS during pregnancy [118,119]; however, large-scale, longitudinal studies are needed to further evaluate safety [120].

Overall, after a full clinical trial and validation, tDCS could potentially become the ideal alternative treatment option for not only depression, but also FM during pregnancy. Additionally, tDCS could be used as an add-on treatment combined with other therapies, such as physical exercise, since non-pharmaceutical interventions have been found to be more beneficial to FM patients than pharmacological treatments [33,121]. Combining exercise with brain stimulation may facilitate neuroplasticity [122]. Moreover, home devices might enable more successful therapeutic applications and make the treatment available to a wider group of patients.

**Table 2:** Studies reporting on efficacy and safety of transcranial direct current stimulation (tDCS) in pregnancy.

	Shenoy et al., 2014 [123]	Sreeraj et al., 2016 [124]	Strube et al., 2016 [125]	Palm et al., 2017 [126]	Vigod et al., 2019 [112]	Kurzeck et al., 2021 [118]
Study Type	Case Report	Case Report	Case Report	Uncontrolled	RCT	Open Label
N	1	1	1	3	20	6
Age	25	23	36	23, 28, 32	>18	23–43
Dropouts	0	0	0	0(1) *	4 **	0(2) *
Diagnosis	AH	MDD	AH	MDD	MDD	MDD
Scale(s)	PSYRATS	HAMD, HAMA	PANSS, AHRS, CDSS, CGI, GAF	HAMD-21, BDI, TMT-A/B	MADRS	HAMD-21, BDI, CGI, TMT-A/B
Treatment	Add-On	Mono	Mono	Mono	Mono	Mono
tDCS prior to Pregnancy	Yes	No	N/A	N/A	N/A	N/A
Weeks in Gestation	18	6	32	19–31	14–32	12–33
Parameters	2 mA, 2 × 20 min	2 mA, 30 min	2 mA, 2 × 30 min	2 mA, 2 × 30 min (2 mA, 30 min)	2 mA, 30 min	2 mA, 2 × 30 min (2 mA, 30 min)
No. of Sessions	10	10	20	20 (30)	15	20 (30)
Response	Near remission	Remission	41% improvement (CDSS)	33.3% remission	75% vs. 12.5%	39.3% reduction (HAMD) 57.1% reduction (BDI) 28.6% reduction (CGI)
Comments	Add-on tDCS resulted in near remission of auditory hallucination. tDCS was well tolerated and no changes in autonomic function, ventilation rate, or core body temperature were observed.	tDCS was well tolerated without any adverse events. In 3 out of the 10 tDCS sessions, patients experienced transient, mild burning sensation at the target side and fleeting perception of phosphenes during the fade-in phase, which is an anticipated tDCS side effect.	No improvement in auditory hallucinations was recorded. Patients tolerated tDCS well with no reported, noticeable side effects. Fetal examination at 35th gestational week revealed no changes or abnormalities. Delivery of a healthy child occurred with no complications.	Statistically significant changes could be observed. One patient achieved remission. tDCS was well tolerated without adverse events.	No abnormalities or serious pregnancy complications were reported in either group. Percent fractions of 87.5% and 77.8% in the tDCS group and sham group, respectively, were satisfied to extremely satisfied with the treatment and viewed tDCS as an acceptable and alternative treatment option.	Significant changes were observed. tDCS was well tolerated without adverse events. In Phase 1, 33.3% achieved response in HAMD scores; 33.3% showed response and 16.7% remission in BDI scores. In Phase 2, one patient achieved remission for both HAMD and BDI.

AH: Auditory Hallucinations; AHRS: Auditory Hallucination Rating Scale; BDI: Beck’s Depression Inventory; CDSS: Calgary Depression Scale in Schizophrenia; CGI: Clinical Global Impression; GAF: Global Assessment of Functioning; HAMA: Hamilton Anxiety Rating Scale; HAMD: Hamilton Depression Rating Scale; MADRS: Montgomery–Asberg Depression Rating Scale; MDD: Major Depressive Disorder; PANSS: Positive and Negative Syndrome Scale; PSYRATS: Psychotic Symptom Rating Scales; RCT: Randomized Clinical Trial; TMT A/B: Trail Making Test A/B. * Patients were submitted to twice-daily tDCS over ten days during inpatient stay, followed by once-daily tDCS over 10 days during an optional outpatient stay. ** One in each group withdrew (1) before the start of the protocol due to an obstetrical complication or childcare challenges and (2) after 1 session both due to travel feasibility.

In summary, the following items should be considered with the aim of developing tDCS neuromodulation treatments for peri-pregnancy FM patients:

- Perform hormonal-related longitudinal studies in FM patients during different hormonal phases: menses, pregnancy, menopause. Progesterone, prolactin, estrogen, and testosterone should be carefully evaluated and matched with symptom fluctuations.
- Further assess the involvement of cortisol and serotonin through repetitive blood and saliva sampling.
- Perform GABAergic investigation using high-resolution functional magnetic resonance imaging (fMRI)/18F-fludeoxyglucose positron-emission tomography (18F-FDG–PET) scans in patients during different hormonal phases would prove how the GABAergic system is modulated [127,128].
- Assess the feasibility of tDCS in women planning a pregnancy affected by FM.
- Assess home-based, remote tDCS treatment in combination with lifestyle changes, given they have been proven to be successful at reducing the patient’s symptoms [33].

## 9. Conclusions

Based on changes in FM symptoms reported by females affected by the disorder during pregnancy, or developing it, we hypothesize that the progressive aggravation towards the delivery is correlated to the negative influence exerted by neuro-endocrine changes on the GABAergic transmission in our previously proposed thalamocortical loop model of FM pathogenesis. This proposes a mechanism of how FM onset occurs during pregnancy, or why recrudescence in late pregnancy occurs in existing FM patients. We suggested new studies be performed to validate our hypothesis. Given the ability of tDCS to modulate the GABAergic system, this evidence merits consideration as a potential treatment option during or before pregnancy. We encourage the scientific community to consider these new perspectives.

## Figures and Tables

**Table 1:** Hormonal reference ranges during menstrual cycle and pregnancy.

		Pregnancy Trimesters		
Hormone	Menstrual Cycle (min and max)	First	Second	Third
Progesterone (ng/mL)	2–25	8–48	32–80	99–342
17β-estradiol (pg/mL)	30–400	188–2497	1278–7192	6137–3460
Prolactin (ng/mL)	< 20	36–213	110–330	137–372
Cortisol (µg/dL)	10–20 (CAR)	7–19	10–42	12–50

Values are from [60,61]. CAR = cortisol awakening response.

## Data Availability

No new data were created in this study. Data sharing is not applicable to this article.
